# Medical Certificates to Assurance Societies

**Published:** 1838-01

**Authors:** 


### MEDICAL CERTIFICATES TO ASSURANCE SOCIETIES.

A great feud has arisen between medical men and the Assurance Societies. A careful man, regardful of advancing time, and the claims of those who will survive him, wishes to secure to them the payment of a certain sum after his death, by the payment of an annual premium as long as he lives. Numerous societies will undertake to do this for him, on the chance of his being likely to live as long as most men of his years. For the purpose of ascertaining this point, important to both parties, the Societies employ and pay a medical referee. Thus far there is nothing objectionable or doubtful. But they also demand and receive the certificate of the good man's medical attendant, while the good man often forgets to pay a fee for it, or offers to pay one and the fee is refused. The medical man cries out against the office, because he gets no fee. Now the question is, is the office in fault? We think not. It is assumed, by those who consider the Office in fault, that the Company is the party which profits, either exclusively or principally. Now this is altogether a mistake, betraying gross ignorance of the principles of assurance. Both parties profit; and, when the Company is founded on sound principles, profit equally. The Company does not seek the party requiring assurance, but the party the Company; and, if the latter does not choose to submit to the regulations of the former, he may refrain from claiming its assistance. A certificate is deemed essential by the Company, and it is incumbent on all claimants of assurance to furnish it. The Company is prepared to pay its own medical officer, when the party comes to be examined: why it should also pay for what is the claimant's affair, not its own, is surely any thing but obvious. We are, therefore, of opinion that medical men are wrong in calling out so loudly for their fees from the Offices; and think that, if they receive them at all, they ought to receive them from the patient who seeks the assurance. Whether such fee should be demanded, or whether this good office should be done gratuitously, is another question, which men will decide according to their feelings. If no visit is required to be paid, and no new examination of the patient is necessary, and all that the physician or surgeon has to do is to fill up the blanks in the ordinary printed circulars, we do think that not only the common rights of citizenship and acquaintanceship, but the honour of the profession and the interest of the professors alike require that no fee should be demanded or received, under such circumstances.

